# Azelnidipine protects myocardium in hyperglycemia-induced cardiac damage

**DOI:** 10.1186/1475-2840-9-82

**Published:** 2010-12-01

**Authors:** Vasundhara Kain, Sandeep Kumar, Amrutesh S Puranik, Sandhya L Sitasawad

**Affiliations:** 1 National Centre for Cell Science, NCCS Complex, Pune University Campus, Ganeshkhind Road, Pune-411007, Maharashtra, India; 2 Interdisciplinary School of Health Sciences, University of Pune, Ganeshkhind, Pune-411007, Maharashtra, India

## Abstract

**Background:**

Azelnidipine (AZL), a long-acting dihydropyridine-based calcium antagonist, has been recently approved and used for treating ischemic heart disease and cardiac remodeling after myocardial infarction, however, its effect on hyperglycemia-induced cardiac damage has not been studied.

**Methods:**

This study examined the effect of AZL on circulating markers of cardiac damage, altered lipid and cytokines profile and markers of oxidative stress including homocysteine in diabetic rats.

**Results:**

STZ induced diabetes caused a significant increase in blood glucose levels. It also resulted in an increase in the levels of homocysteine and cardiac damage markers, like Troponin-1, CK-MB, CK-NAC, uric acid, LDH and alkaline phosphatase. Moreover, there was an increase in the levels of proinflammatory cytokines like TNF-α, IFN-γ, and TGF-β and decrease in the levels of IL-4 and IL-10. Additionally, there was increase in the levels of cholesterol, triglycerides, LDL, VLDL and a decrease in HDL in these animals. There was an altered antioxidant enzyme profile which resulted in a notable increase in the levels of oxidative stress markers like lipid peroxides, nitric oxide and carbonylated proteins. Compared with the untreated diabetic rats, AZL treatment significantly reduced the levels of troponin-1 (P < 0.05), CK-MB (P < 0.05), CK-NAC (P < 0.05), uric acid (P < 0.05), LDH (P < 0.05) and alkaline phosphatase (P < 0.05). It also reduced the levels of the TNF-α (P < 0.05), IFN-γ (P < 0.05), and TGF-β (P < 0.05) and increased the levels of IL-4 (P < 0.05). A significant decrease in the serum cholesterol (P < 0.05), triglycerides (P < 0.05), LDL (P < 0.05), VLDL (P < 0.05) and a significant rise in levels of HDL (P < 0.05) was also observed. Treatment with AZL corrected the distorted antioxidant enzyme profile resulting in a significant decrease in the levels of lipid peroxides, nitric oxide and carbonylated proteins.

**Conclusion:**

Our results indicate that AZL treatment can reduce the risk of hyperglycemia induced metabolic disorders and its role can be further extended to explore its therapeutic potential in diabetic patients with cardiac complications.

## Background

Improper management of metabolic disturbances especially altered lipid and protein metabolism in diabetes results in grave cardiovascular complications which causes increasing diabetes-related mortalities [1]. Dyslipidemia, oxidative stress and inflammatory injury are important interrelated factors responsible for the development of cardiomyopathy as they are known to promote the progression of premature atherosclerosis, coronary insufficiency and myocardial infarction [2]. Various studies have correlated that the circulating markers of myocardial damage, pro-inflammatory cytokines, calcium dysregulation, oxidative stress and atherogenic lipids are elevated in diabetic patients [3-10].

Metabolically, the diabetic heart is characterized by diminished glucose utilization and increased fatty acid oxidation resulting in lipid accumulation in the myocardium [11,12]. This myocardial lipotoxicity results in alterations in the inflammatory cytokines levels and evokes a cascade of disparaging changes that leads to cardiac damage. Recently, proinflammatory cytokines like TNF-α and IFN-γ have been shown to alter the calcium regulation and directly or indirectly reduce the myocardial contractility [13-18]. Likewise, the proinflammatory and profibrotic cytokine, TGF-β contributes to the *in vivo *cardiac electrical remodeling by decreasing cardiac muscle L-type calcium current and charge movement [19,20]. These cytokine-induced cascades of events mechanistically contribute to cardiomyocyte apoptosis possibly due to disturbance of calcium homeostasis via redox regulatory mechanisms leading to extensive myocyte cell death.

There is an alteration in the antioxidant enzyme levels which leads to oxidative stress as the production of reactive oxygen species (ROS) exceeds its scavenging. Oxidative stress directly leads to lipid peroxidation, protein carbonylation and cardiac fibrosis significantly contributing to pathophysiology of cardiac complications of diabetes [21-25]. It has been shown that increased calcium flux further aggravates oxidative stress and vice versa [26-29]. Thus the role of calcium channel blockers (CCBs) becomes extremely important for correcting this underlying phenomenon.

CCBs initially used to treat hypertension found additional beneficial implications in various conditions, such as angina pectoris, hypertrophic cardiomyopathy, pulmonary hypertension and diabetic cardiomyopathy [30]. There are 3 main classes--phenylalkylamines, benzothiazepines, and dihydropyridines (DHP) because of difference in their molecular structure, sites and modes of action, and effects on various other cardiovascular functions [31]. Primarily, the CCBs lower blood pressure through vasodilation and reduction of peripheral resistance and usually do not impair glucose metabolism or lipid profile and may even attenuate the development of arteriosclerotic lesions [30]. Inferring from these studies, it seems that calcium antagonists are also safe and effective as first-line or add-on therapy in diabetic hypertensive patients [32]. Thus, CCBs with lipid-lowering, anti-oxidative and/or anti-inflammatory activities may potentially prevent or delay the occurrence of diabetic cardiomyopathy.

AZL, a newly developed and commercially used novel long-acting DHP-based calcium antagonist has been reported to be effective in treating ischemic heart disease and cardiac remodeling after myocardial infarction (MI) [3] and reduce blood pressure without increasing the heart rate in patients with hypertension [33,34]. In the experimental animals, AZL revealed anti-atherosclerotic effects independent of its blood pressure-lowering actions [35]. In another study, AZL prevented the TNF-induced endothelial cell activation via its antioxidative properties [36]. AZL also inhibited H_2_O_2_-induced cell death in neonatal rat cardiomyocytes [37]. However, the effects of AZL on diabetes induced cardiac damage have not been studied.

In the light of the above findings, we chose to investigate the possible effect of AZL on the circulating markers of cardiac damage (troponin 1, CK-MB, CK-NAC, LDH, uric acid), oxidative stress (lipid peroxides, nitric oxide, carbonyl content), antioxidant enzyme (superoxide dismutase, catalase and reduced glutathione), serum hcy, proinflammatory and anti-inflammatory cytokines (TNF-α, IFN-γ, TGF-β, IL-4 and IL-10) and atherogenic lipid profile in STZ induced diabetic rats.

## Methods

Six to eight-week-old male Wistar rats (NCCS, Pune, India) weighing 300 to 330 g were made diabetic by a single intraperitoneal injection of streptozotocin (STZ) (55 mg/kg, Sigma, St. Louis, MO) as described previously [23]. Control animals were treated with vehicle (0.1 mol/L sodium citrate buffer, pH 4.5). Hyperglycemia (blood glucose > 200 mg/dL) was confirmed 3 days post STZ injection using a glucometer (AccuCheck; Roche, Germany). Diabetic animals were treated with AZL suspended in 1% carboxy methyl cellulose at a single daily dose of 5 mg/kg, administered orally by gavage on the 4^th ^day of STZ treatment (*n *= 12) for 12 weeks. At the end of the study duration, rats were fasted overnight, anesthetized using 500 mg/kg dose of urethane and the blood was collected via heart puncture with a 19½ gauge needle. The animals were then euthanized by cervical dislocation. Urethane was selected in the present study as an anesthetic agent as a single dose induces long term anesthesia with minimal cardiovascular and respiratory system depression. It is a convenient one shot method for non-recovery studies and produces a level of surgical anesthesia and analgesia characterized by preservation of a number of cardiovascular reflexes [38]. All procedures were approved by Institutional Animal Care and Use Committee and complied with standards for the care and use of animal subjects, as stated in the *Guide for the Care and Use of Laboratory Animals *(Institute of Laboratory Resources, National Academy of Sciences, Bethesda, MD).

Heart was excised and the heart weight (HW) and left ventricular mass (LVM) was noted.

For the isolation of plasma, the blood was collected in EDTA-coated *Vacutainer *(BD *Vacutainer*^®^, USA) was isolated after centrifuging blood in a pre-cooled centrifuge at 1500 rpm for 10 min at 1500 × g. For isolation of serum, the blood was allowed to clot at room temperature for 15 to 20 minutes then centrifuged for 10 minutes at 1000 × g.

### Serum Insulin

Serum insulin levels were measured by the High-range Rat Insulin ELISA kit from Mercodia AB, (Sweden) as per manufacturer's instructions[39].

### Quantification of hcy levels in the serum by High Performance Liquid Chromatography (HPLC)

Serum hcy was analyzed by HPLC as described previously [40]. The HPLC system (Dionex, Germany) consisted of P-680 quaternary gradient pump, an ASI 100 autosampler, a universal chromatographic interface UCI-50 and fluorescence detector RF-2000 integrated by Chromeleon Software 6.70 coupled to pH stable reverse phase C18 column (4.6 × 250 mm, Varian, Varian Inc). Thiol-specific fluorogenic reagent 4-(aminosulfonyl)-7-fluoro-2,1,3-benzoxadiazole (ABD-F) was used as flurochrome. Mobile phase used was ammonium phosphate buffer (0.01 M) and acetonitrile (80:20) with flow rate as 0.5 mL per min. The limit of detection of hcy (Sigma Inc, USA) was 500 nmol L^-1^.

### Cytokines assay

TNF-α, IFN-γ, TGF-β, IL-4 and IL-10 in the plasma were determined by the sandwich ELISA method using a commercially available kit from BD OptEIA ELISA Set (BD Biosciences, USA) as per manufacturer's instructions. The data are expressed as picogram per milliliter. For each assay the cytokine standards were used each time to check the variation from plate to plate on different days of analyses.

### Biochemical Markers of Myocardial Injury in serum

Estimation of total cholesterol, low density lipoprotein, high density lipoproteins, very low density lipoproteins, creatine kinase (CK-MB and CK-NAC), lactate dehydrogenase (LDH), uric acid and troponin I in the serum was done using appropriate kits on RA-50 semi auto analyzer by well standardized methods.

### Liver function tests

Quantitative estimation of Alanine aminotranferease (ALT), aspartate aminotransferase (AST) and alkaline phosphatase was done using specific kit [Erba Diagnostics Mannheim GmBH, Germany] using RA -50 semi auto analyzer by well standardized methods [41].

### Mesurement of lipid peroxidation

Determination of lipid peroxidation (nmole malondialdehyde/mg protein) was done spectrophotometrically using the thiobarbituric acid-reactive substance (TBARS) by the method of Uchiyama and Mihara [42] using 1,1,3,3-tetramethoxypropane as a standard.

### Estimation of antioxidant enzyme levels

The level of SOD and catalase enzyme activities was measured using the SOD Assay Kit-WST and Catalase Assay kit (Sigma, St. Louis, MO) according to the manufacturer's instructions. The concentration of reduced glutathione (GSH) was determined according to Ellman [43]. All assays were performed in triplicate and on the same day to reduce inter-assay variation. Activities were normalized to protein in the sample using previously described spectrophotometric dye binding methods [44].

### Estimation of Nitrite oxide

Nitric oxide was quantitated by using Griess Reagent Kit for Nitrite Determination (Molecular Probes, USA) as per manufacturer's instructions.

### Protein carbonyl assay

The level of protein carbonyl groups was estimated according to the method of Levine et al. [45] with slight modifications. Briefly, the samples were incubated with 2,4-dinitrophenylhydrazine (DNPH) for 1 h followed by precipitation with trichloroacetic acid. After centrifugation, the pellets were washed with an ethanol-ethyl acetate mixture to remove the excess DNPH, dissolved in guanidine hydrochloride solution (6 mol/L) and the absorbance was measured at 370 nm. The content of carbonyl groups was calculated using the molar extinction coefficient of 22,000 (l/cm mol). The content of protein was assayed by the method of Lowry et al [46], using bovine serum albumin as standard.

### Statistical analysis

Data are expressed as means ± standard error (SE) and were analyzed using one-way analysis of variance and secondary analysis for significance with Tukey-Kramer post tests using Prism 4.0 GraphPad software (GraphPad, *San Diego*, CA, USA). A *p *value less than 0.05 was considered statistically significant.

## Results

### Characterization of animal groups

Animals in the untreated STZ diabetic group displayed severe hyperglycemia, significant decrease in the levels of insulin and an increase in the LVM/BW ratio (as an index of ventricular hypertrophy). AZL treatment reduced the glucose levels although the levels remained elevated than the controls. AZL treatment also improved the levels of serum insulin. AZL treatment resulted in a significant decrease in the LVM/BW ratio indicating that AZL treatment prevents left ventricular hypertrophy. The data for blood glucose, serum insulin, and LVM/BW ratio has been tabulated in Table 1. There was also a notable increase in the levels of total serum cholesterol, triglycerides, LDLs and VLDL. Levels of HDL decreased in the STZ-diabetic rats there by significantly increasing the total cholesterol to HDL ratio and HDL to LDL ratio.

**Table 1:** Effect of AZL treatment on blood glucose, insulin, homocysteine and LVW/BW ratio in STZ-treated diabetic rats.

Parameters	Control (n = 10)	STZ* (n = 10)	AZL (5 mg/kg/day)** (n = 10)
Blood Glucose (mg/dL)	167.5 ± 25.5	517.5 ± 45.5	256.2 ± 12.4
Serum Insulin (ng/mL)	3.3 ± 0.4	0.42 ± 0.3	1.40 ± 0.4
Homocysteine (μM/L)	2.9 ± 0.7	26.5 ± 4.4	13.8 ± 3.9
LVW/BW ratio (mg/g)	3.5 ± 0.12	4.9 ± 0.02	3.4 ± 0.43

Values are mean ± SE. * means p < 0.05 compared to control group, ** means p < 0.05 compared to STZ diabetic group.

### AZL treatment decreases the levels of Hcy

Hcy concentration in blood has been predicted as a powerful prognostic marker of cardiac complications of diabetes [47]. STZ diabetic rat showed a significant increase in the levels of hcy which were significantly reduced after AZL treatment (Table 1).

### AZL treatment reduces the markers of cardiac damage

**Figure 1:**
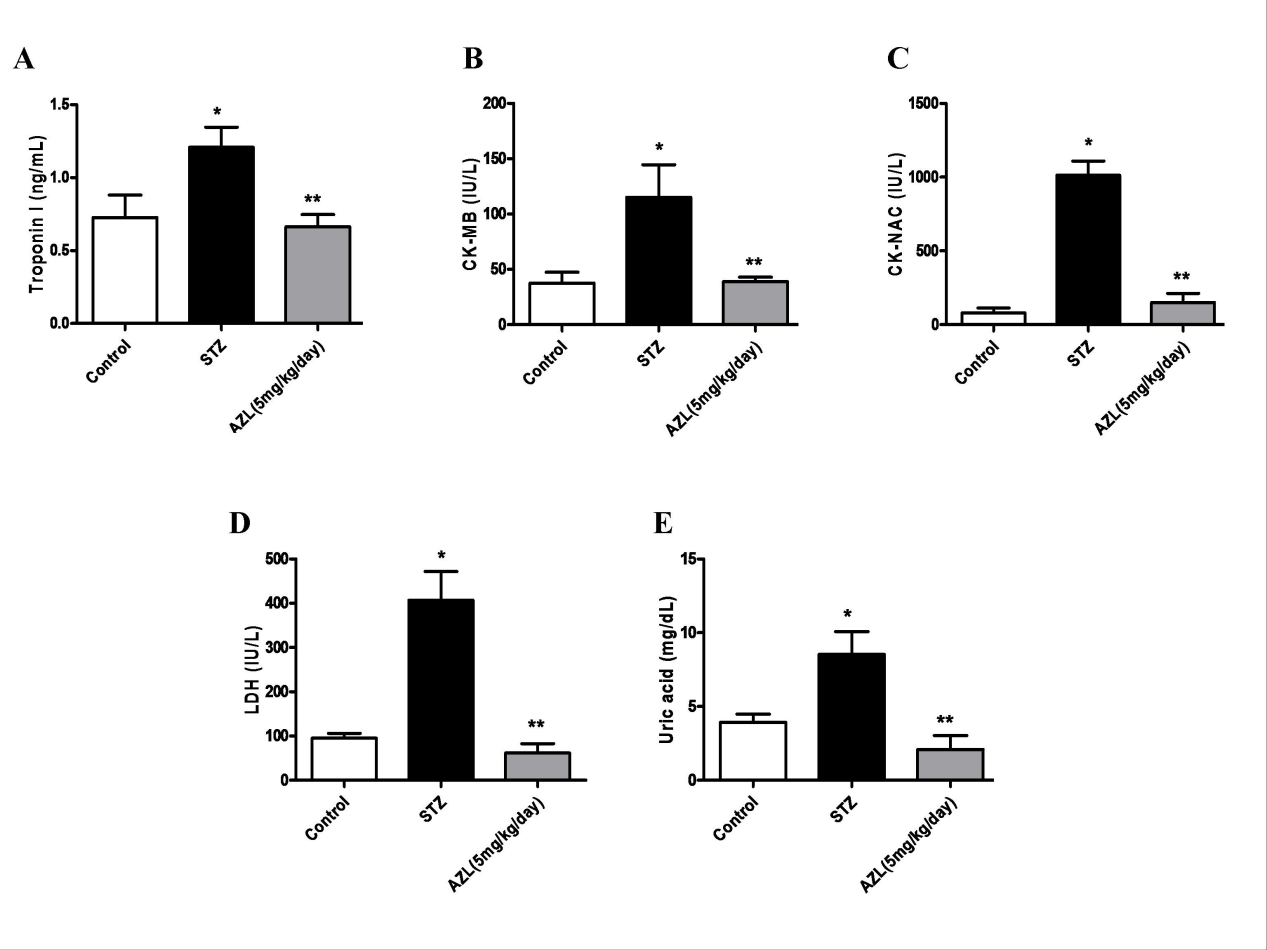
**Effect of AZL treatment on serum cardiac markers in STZ-treated diabetic rats (A) Troponin I (B) CK-MB (C) CK-NAC (D) LDH (E) Uric acid levels**. Values depict means ± SE for at least n = 8 animals in each group. * means p < 0.01 compared to control group and ** means p < 0.01 compared to STZ diabetic group

Compared to the controls, the circulating markers of cardiac damage in STZ diabetic rat, showed a significant increase in the levels of serum troponin I (1.21 ± 0.13 ng/mL vs. 0.72 ± 0.15 ng/mL), CK-MB (135.0 ± 29.5 IU/L vs. 37.50 ± 9.9 IU/L) and CK-NAC (1013.0 ± 96.3 IU/L vs. 81.21 ± 32.43 IU/L), LDH (407.0 ± 64.72 IU/L vs. 95.0 ± 10.99 IU/L) and uric acid (8.53 ± 1.54 mg/dL vs. 3.93 ± 0.5 mg/dL). This notable increase was significantly reduced by AZL treatment. The levels of troponin I (0.66 ± 0.08 ng/mL), CK-MB (38.8 ± 4.0 IU/L), and CK-NAC (150.32 ± 61.2 IU/L), LDH (61.50 ± 21.0 IU/L), Uric acid (2.1 ± 0.93 mg/dl) and alkaline phosphatase (149.6 ± 8.43 IU/L) were significantly lower when compared with the STZ diabetic group (Figure 1).

### Improvement of proinflammatory cytokines after AZL treatment

**Figure 2:**
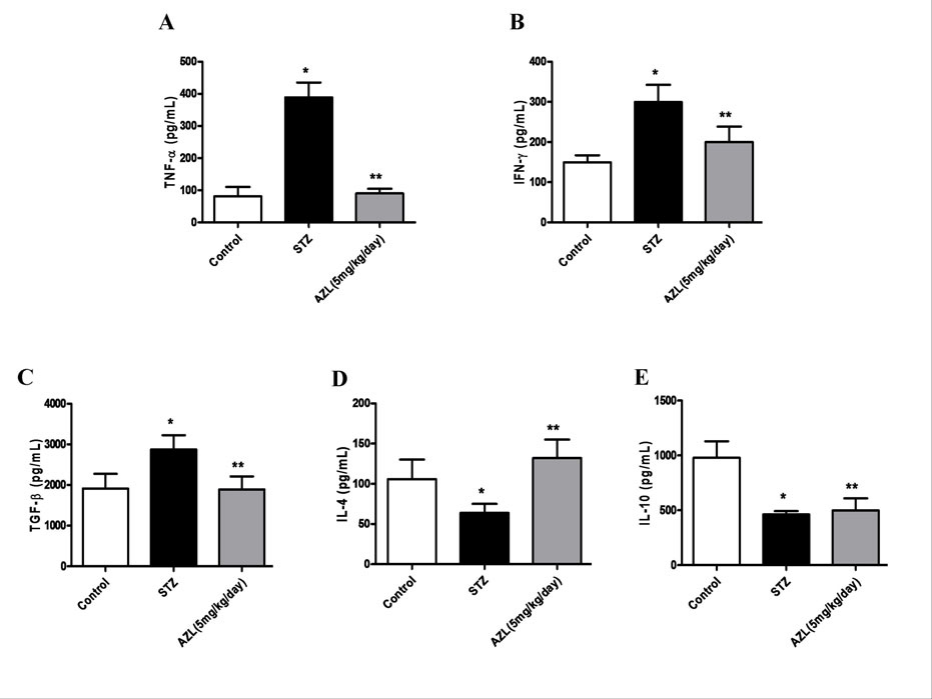
**Effect of AZL treatment on plasma cytokine profile in STZ-treated diabetic rats**. (A) TNF α (B) IFN-γ (C) TGF-β (D) IL-4 (E) IL-10 levels. Values depict means ± SE for at least n = 8 animals in each group. * means p < 0.001 compared to control group and ** means p < 0.01 compared to STZ diabetic group.

Compared to the controls, STZ diabetic rat demonstrated significantly increased levels of the pro-inflammatory cytokines TNF-α (389.1 ± 46.2 pg/mL vs. 81.6 ± 28.3 pg/mL), IFN-γ (299.3 ± 42.9 pg/mL vs. 149.6 ± 17.6 pg/mL), TGF-β (2875.3 ± 349.08 pg/mL vs. 1913.3 ± 362.1 pg/mL). There was a notable decrease in levels of IL-4 (63.8 ± 11.1 pg/mL vs. 105.9 ± 24.2 pg/mL) and IL-10 (462.4 ± 31.2 pg/mL vs. 979.5 ± 150.3 pg/mL). AZL treatment prevented the increase in the levels of TNF-α (90.0 ± 14.8 pg/mL), IFN-γ (200.2 ± 38.1 pg/mL), and TGF-β (1892.4 ± 321.0 pg/mL). It also resulted in an increase in the levels of IL-4 (132.1 ± 22.9 pg/mL), which was even higher than the controls (Figure 2). However, levels of IL-10 remained unchanged (499.2 ± 110.5 pg/mL) in AZL treatment group when compared to STZ diabetic group.

### Normalization of altered lipid profile after AZL treatment

AZL had a significant effect on lowering triglyceride, cholesterol, LDL and VLDL levels and increasing HDL levels in diabetic rats (Table 2A). Diabetic rats treated with AZL also showed significant decreases in total cholesterol and total cholesterol/HDL ratio, as well as elevated levels of HDL cholesterol (Table 2B). All these levels were completely normalized after AZL treatment which suggests that AZL is far more effective in maintaining the lipid profile near to that of control in this animal model of diabetes.

**Table 2:** (A) Effect of AZL treatment on serum lipid profile. B) Atherogenic risk marker and cardiovascular risk indicator.

	CTRL	STZ	AZL(5 mg/kg/day)
SERUM CHOLESTEROL (mg/dL)	70.3 ± 6.4	165 ± 9.6*	71 ± 5.9^#^
SERUM TRIGLYCERIDES (mg/dL)	68 ± 7.4	146 ± 12.5*	74 ± 11.3^#^
HDL (mg/dL)	46 ± 3.5	29 ± 7.1*	48 ± 5.2^#^
LDL (mg/dL)	34 ± 3.9	66 ± 7.9*	41 ± 5.0^#^
VLDL (mg/dL)	13.6 ± 3	29.2 ± 5.5*	14.8 ± 5.1^#^
Total Cholesterol/HDL	1.5 ± 0.2	5.6 ± 0.1*	1.4 ± 0.2^#^
LDL/HDL	0.7 ± 0.1	2.2 ± 0.1*	0.8 ± 0.1^#^

Values depict mean ± SE for at least n = 6 animals in each group. * means p < 0.05 compared to control group and # means p < 0.05 compared to STZ diabetic group.

### Effect of AZL on the markers of liver function

**Figure 3:**
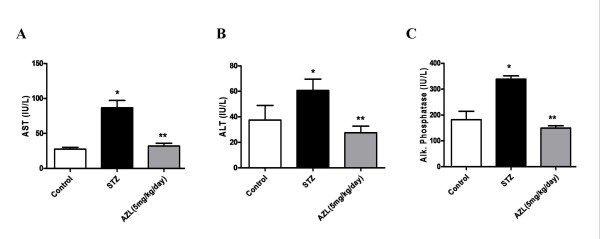
**Effect of AZL treatment on liver function test (A) AST (B) ALT (C) Alkaline phosphatase in serum of STZ-treated diabetic rats**. Values depict mean ± SE for at least n = 8 animals in each group. * means p < 0.001 compared to control group and ** means p < 0.01 compared to STZ diabetic group.

Diabetes was found to increase the levels of the liver marker enzymes ALT (60.7 ± 8.8), AST (86.7 ± 10.5) and alkaline phosphatase (330.0 ± 12.51 IU/L) compared with those of the controls viz. 37.5 ± 11.5, 27.7 ± 2.5 and 181.7 ± 32.9 IU/L, respectively. After treatment with AZL, the levels of ALT, AST and alkaline phosphatase were observed to be normalized at 27.5 ± 5.2 for ALT, 32.0 ± 4.0 for AST and 149.6 ± 8.4 IU/L for alkaline phosphatase, indicating that AZL treatment does not metabolically induce xenobiotic hepatotoxicity (Figure 3).

### Effect of AZL treatment on diabetes-Induced Oxidative Stress

**Figure 4:**
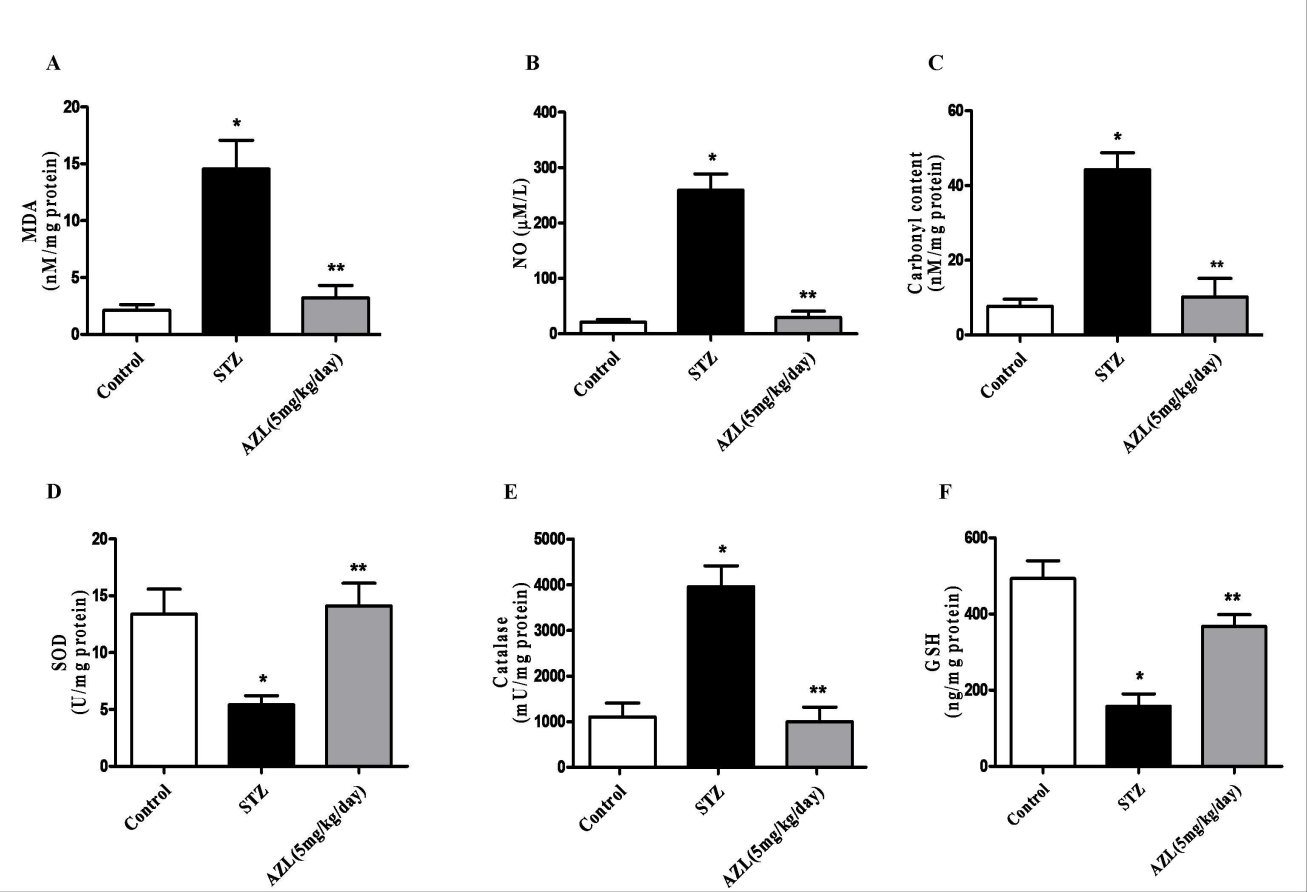
**Effect of AZL treatment on levels of oxidative stress markers and antioxidants**. (A) Lipid peroxidation (B) Nitric oxide (C) Carbonyl content (D) Superoxide dismutase (E) reduced glutathione (F) Catalase. Values are means ± SE of 6 rats. * means p < 0.001 compared to control group, ** means p < 0.01 compared to STZ diabetic group.

Lipid peroxides formation (nmol MDA/mg protein) which is a direct marker for oxidative stress increased significantly in the cardiac tissue of STZ group compared with the control (STZ 14.5 ± 2.5 vs. control 2.1 ± 0.5, *P *< 0.05). AZL treatment significantly reduced the levels of lipid peroxidation (3.2 ± 1.1, *P *< 0.05) which was very close to the control group (Figure 4A). With respect to the control group the levels of carbonyl content (nmol/mg protein), another indicator of oxidative stress, were also increased in diabetic rat (STZ 44.2 ± 4.5 vs. control 7.7 ± 1.9, *P *< 0.05) and AZL treatment significantly prevented this increase (10.2 ± 4.9, *P *< 0.05) (Figure 4B). Levels of nitric oxide, which is indicative of nitrosative stress (μmol/L), increased significantly in diabetic rat heart when compared to control (STZ 259.3 ± 29.3 vs. control 21.3 ± 4.4, *P *< 0.05). This nitric oxide level after AZL treatment were reduced (29.55 ± 11.3, *P *< 0.05) and were close to control group (Figure 4C).

### Effect of AZL treatment on antioxidant enzyme levels

Levels of SOD (U/mg protein) and GSH (ng/mg protein) were depressed significantly in the diabetic rat (STZ 40 ± 0.8 vs. control 13.4 ± 2.2, *P *< 0.05 and GSH 57.9 ± 32.3 vs. control 493.5 ± 46.2, *P *< 0.05), whereas level of catalase (mU/mg protein) increased significantly compared with controls (STZ 3955.6 ± 457.3 vs. control 1102.8 ± 304.3, *P *< 0.05). These different enzyme and non-enzyme antioxidant levels were normalized after AZL treatment (SOD 14.1 ± 2.0, *P *< 0.05; GSH 367.26 ± 31.1, *P *< 0.05 and catalase 996.65 ± 322.60, *P *< 0.05) (Figure 4D-F).

## Discussion

Diabetes is a metabolic syndrome with a cluster of common clinical disorders that is related with an increased risk for cardiovascular disease [48]. Despite some controversy in the past [49], large patient studies have demonstrated that calcium channel blockers are effective in reducing the mortality and morbidity of cardiovascular disease [50,51]. AZL is one of the cardiobeneficial CCB that decreases blood pressure without increasing the heart-rate [33,34], prevents cardiac damage [3], reduce glucose intolerance [52] and possesses antioxidant properties [37]. However, there is a significant gap in our knowledge about the cardioprotective action and efficacy of AZL as the effect of AZL on proinflammatory cytokines and altered lipid profile in either diabetic patients or in experimental models of diabetes has not been studied so far. In the present study, we extensively evaluated the effect of AZL on circulating markers of cardiac damage secondary to diabetes.

Diabetic cardiomyopathy characterized by diastolic dysfunction and left ventricular hypertrophy is usually the terminal condition of heart in diabetes [53]. Our results indicate that 12-week treatment of AZL in diabetic rats proved to be beneficial as it restrained the progression of the metabolic disorders of diabetes. The STZ diabetic rats showed elevated blood glucose levels with reduced levels of serum insulin. Also, there was a notable increase in the LVW/BW ratio which signifies cardiac hypertrophy. AZL did not affect the serum insulin levels indicating that it does not affect the function of β-cells in the pancreas although there was a remarkable improvement in the blood glucose levels. AZL has been shown to improve glucose intolerance in the diabetic mice mainly through inhibition of oxidative stress [52]. This improvement in the blood glucose levels could be attributed to its ancillary properties partially because of its lipid lowering capability and partially because of its antioxidant nature. AZL treatment showed a distinct positive effect on LVW/BW ratio indicating that AZL was able to prevent cardiac hypertrophy which usually sets in as a result of diastolic dysfunction secondary to diabetes.

Elevated hcy level is also an independent risk factor for the development of atherosclerosis as well as a prognostic marker in ischemic heart disease [47,54]. Protein homocysteinylation is a novel example of protein damage that can be attributed to hyperhomocysteinemia [55]. Homocysteinylated proteins were prone to multimerization and underwent gross structural changes that led to their denaturation. Homocysteine thiolactone may also inactivate enzymes responsible for posttranslational modification of connective tissue matrices leading to fibrosis and scarring [56]. Hcy also promotes atherogenesis through oxidative damage that is mediated by cytotoxic free radicals or by the induction of pro-inflammatory factors [57]. Moreover, under the diabetic condition, endogenously upregulated homocysteine levels induce endothelial-myocyte uncoupling which further aggravate the deteriorating condition of the myocardium [58]. In our study, AZL treatment significantly lowered the elevated Hcy levels indicating that AZL can prevent accumulation of free radicals, atherogenic plaque formation and reducing protein homocysteinylation.

Diabetes is associated with an increase in the oxidative stress that results from exhaustion of cellular antioxidants and depletion of antioxidant enzymes [59]. The results from our study showed that STZ induced diabetic condition resulted in an increase in the lipid peroxidation and protein carbonylation which are direct indicators systemic oxidative stress. This was in conjunction with depletion of superoxide scavenger SOD and reduced glutathione along with an increase in the levels of antioxidant enzyme catalase. AZL treatment in the STZ-diabetic rats reduced the formation of lipid peroxides, nitric oxides and carbonyl content and also restored the levels of SOD and GSH. AZL also reduced the levels of catalase indicating a reduction in the accumulation of reactive oxygen species. This positive beneficial effect of AZL could be directly attributable to its antioxidative nature which is agreement with previous findings [52,58].

It is widely accepted that high levels of circulating cardiac damage markers like troponin I, CK-MB, CK-NAC, LDH and uric acid represent a powerful and sensitive predictor of increased cardiac complications [60]. In this study, AZL-treated diabetic rats showed a significant improvement in levels of these circulating cardiac damage markers. This indicates that AZL prevents cardiac damage and has beneficial properties far beyond its antihypertensive actions. One of the possible mechanisms of prevention may partially be related to suppression of severe increase in the levels of pro-inflammatory cytokines which was observed in STZ diabetic rats. Our results were in agreement with previous reports where AZL treatment prevented cardiac dysfunction in patients with essential hypertension [34]. This decrease in the cardiac damage markers in the diabetic rats upon AZL treatment supported the hypothesis that AZL reduce the risk of metabolic disorders associated with diabetic. Furthermore, there was no change in serum markers of liver function in AZL treated group compared with untreated diabetic rats, which suggests that AZL treatment did not cause any xenobiotic side effects during the study.

Apart from the altered metabolic condition, diabetes is also an inflammation-prone condition. Hyperglycemia-induced ROS stimulates signal transduction to instigate inflammatory cytokines, e.g. TNF-α, IFN-γ and TGF-β [61]. This leads to systemic inflammation, cardiac dysfunction and exacerbates the severity of diabetes [62]. In the present study, AZL-treated diabetic rats showed effective suppression of these pro-inflammatory cytokines. These results strongly indicate that AZL is a potential agent against diabetes-associated systemic inflammation. We also noticed that AZL increased the levels of the anti-inflammatory and immunosuppressive cytokine IL-4 but did not affect the levels of IL-10. Thus, the resulting reduction in the inflammation in the AZL treated group was due to the stimulation of IL-4 but probably not due the production of anti-inflammatory cytokine IL-10. Inhibition of circulating levels of TNF-α, IFN-γ and TGF-β and an increase in the levels of IL-4 can explain the lowering of serum triglyceride and total cholesterol levels, potentially mediated, at least in part, by the increased glucose sensitivity and glucose metabolism in AZL-treated diabetic rats.

In our study, the altered lipid profile observed in the diabetic condition was also abrogated by AZL treatment. AZL improved the serum cholesterol, triglycerides, LDL and VLDL. It also improved the markers of atherogenic risk, (total cholesterol/HDL and LDL/HDL ratio). These findings suggest that AZL possesses lipid lowering activities due to its unique anti-atherogenic properties which also contribute additionally to its cardiovascular protective actions. This is the first report which evaluated the positive effect of AZL on lipid profile in STZ-diabetic rats *Diabetes Care***27Full Text**.

## Conclusion

In conclusion, we showed that AZL treatment abrogated the metabolic disorders in STZ-diabetic rats at least partly through its antioxidant and antiatherogenic properties. These results indicate that the protective effect of AZL might be, at least in part, due to its inhibitory ability on free radical formation, or due to its free radical scavenging ability. These data also indicate that AZL exerts cardioprotection due to its unique modulatory and antioxidative property and thus may be a promising 'cardioprotective DHP' that can even target the secondary cardiac complication of diabetes. This property of AZL could be further extended to clinics and can be used as adjuvant therapy in cardiac complications especially in diabetes.

## Competing interests

The authors declare that they have no competing interests.

## Authors' contributions

VK and SK contributed equally to the experimental designing and bench work. ASP performed the Hcy estimation and SLS conceived and designed the study. All authors have read and approved the final manuscript.
